# A Cellular and Transcriptomic Atlas of the Aged Mouse Hematopoietic System

**DOI:** 10.1111/acel.70394

**Published:** 2026-01-28

**Authors:** Ryan R. White, Kun Xiong, Matthew Wakai, Allison Surian, Christina Adler, Nicole Negron, Min Ni, Tea Shavlakadze, Yu Bai, David J. Glass

**Affiliations:** ^1^ Aging/Age‐Related Disorders Regeneron Pharmaceuticals Tarrytown New York USA; ^2^ Molecular Profiling Regeneron Pharmaceuticals Tarrytown New York USA

## Abstract

Aging is a dominant risk factor for chronic diseases characterized by the functional decline of tissues and organs. During aging, the hematopoietic system declines in regenerative capacity—seemingly attributable to increases in DNA damage, replicative stress, and autophagic flux—resulting in skewing towards a myeloid lineage and away from a lymphoid lineage. Here, we characterized the transcriptomic and cellular landscape of the aged C57Bl/6J mouse hematopoietic system using a combination of bulk RNAseq and single cell RNAseq (scRNAseq). We show that aging leads to global transcriptional alterations in bulk peripheral blood mononuclear cells (PBMCs), lineage marker‐depleted bone marrow cells (Lin‐BM), and in hematopoietic stem and progenitor cells (HSPCs), immunophenotypically lineage marker negative (Lin‐) Sca1+ cKit+ (LSK+). These changes indicate widespread activation of inflammatory processes, namely in PBMCs and Lin‐BM cells. Interestingly, there is also a downregulation of cell cycle genes in HSPCs during aging. ScRNAseq across 39 hematopoietic cell types revealed age‐related skewing in cell composition. Aged PBMCs showed significant decreases in CD4 and CD8 naïve cells concomitant with increases in CD4/8 memory and CD8 exhausted T cell populations. Lin‐BM cells showed significant myeloid skewing in common myeloid progenitor (CMP) cells, as well as in the HSC population. We also identified a unique HSC population marked by increased *Vwf, Wwtr1*, and *Clca3a1* expression that does not exist in young HSCs, thus likely marking true aged HSCs. Collectively, this work should serve as a useful resource for understanding and therapeutically targeting the aged hematopoietic system.

## Graphical abstract

Transcriptomic and cellular atlas of peripheral blood mononuclear cells (PBMCs) and stem and progenitor cells of the hematopoietic compartment (HSPCs) of aged C57Bl/6J mice. Analysis shows global alterations related to immune function and identifies potential pathways and cell populations that can be targeted for therapeutic intervention.

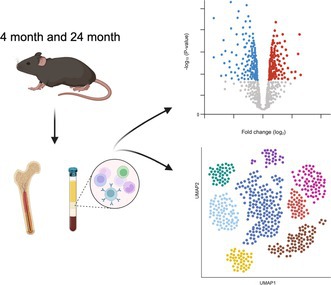

## 1 Introduction

The hematopoietic system is highly regenerative and can be fully reconstituted from a single hematopoietic stem cell (HSC), giving rise to both myeloid and lymphoid lineages (Osawa et al. 1996). During aging, however, HSCs decline in regenerative capacity—likely attributable to an increase in DNA damage and/or an increase in replicative stress (Morrison et al. 1996; Warren and Rossi 2009; Beerman et al. 2010). DNA damage and the subsequent accumulation of mutations in HSCs over time is almost certainly the prime cause for the increased incidence of clonal populations within the mosaic blood compartment (Rossi et al. 2007; Beerman et al. 2014), transitioning from a polyclonal to an oligoclonal state (Ganuza et al. 2019; Mitchell et al. 2022), giving rise to what is now termed clonal hematopoiesis of indeterminate potential (Kessler et al. 2022).

In parallel to clonal diversity declining with age, there is also the well‐documented deterioration of the hematopoietic system with age, coined immunosenescence (Walford 1964; Franceschi et al. 2000). During this process the blood system fails to respond properly to external and internal stimuli, that is, loss/failure of the adaptive immune response (Nikolich‐Zugich 2008). While immunosenescence itself may contribute to the increase in infections observed in the elderly, the inherent increase in senescent, that is, permanently cell cycle arrested, circulating blood cells in the body has also been shown to drive peripheral tissue aging through non‐cell autonomous mechanisms (Yousefzadeh et al. 2021). Moreover, it has also been shown that there is a proportional decrease in classical naïve T cells that may be able to mount a proper immune response even while total lymphocyte numbers are increased (Lin et al. 2016; Krishnarajah et al. 2022). Studies have also shown shifts in HSC gene expression and protein content with age that led to delayed or terminally paused differentiation (Hennrich et al. 2018; Zaro et al. 2020; Herault et al. 2021). These changes are concurrent with the increase in HSC pool and its myeloid lineage bias with age, potentially governed by the increase in expression of the genes *VWF*, *CD150*, and *TAZ* (Zaro et al. 2020; Kim et al. 2022).

While many studies aimed to study either HSCs or PBMCs, none have aimed to characterize the entire hematopoietic system in a collective manner (Zaro et al. 2020; Herault et al. 2021; Zhu et al. 2023; Filippov et al. 2024). For example, we recently published a study, focusing in particular on murine T cells, including multiple time points throughout the lifespan of the lab mouse, to get a thorough view of the pattern of age‐related gene expression changes occurring during aging (He et al. 2025). Previous studies of the various hematopoietic system transcriptomes, and even single‐cell analyses, have been limited by sample size, number of cells/populations analyzed, or were overly reliant on complex sorting strategies (Solomon et al. 2020; Herault et al. 2021; Krishnarajah et al. 2022; Zhou and Cao 2025). Prior limitations in subject number are of particular concern, since low N RNAseq analysis has been shown to lead to a high percentage of false positive findings and a lack of discovery of “real hits” (Halasz et al. 2025). We therefore sought to fully characterize the transcriptomic landscape of the aged mouse hematopoietic system using a combination of bulk RNAseq and single cell RNAseq (scRNAseq) in both the peripheral blood and bone marrow niche.

## 2 Results

### 2.1 Phenotyping of Aged Hematopoietic System

**FIGURE 1:**
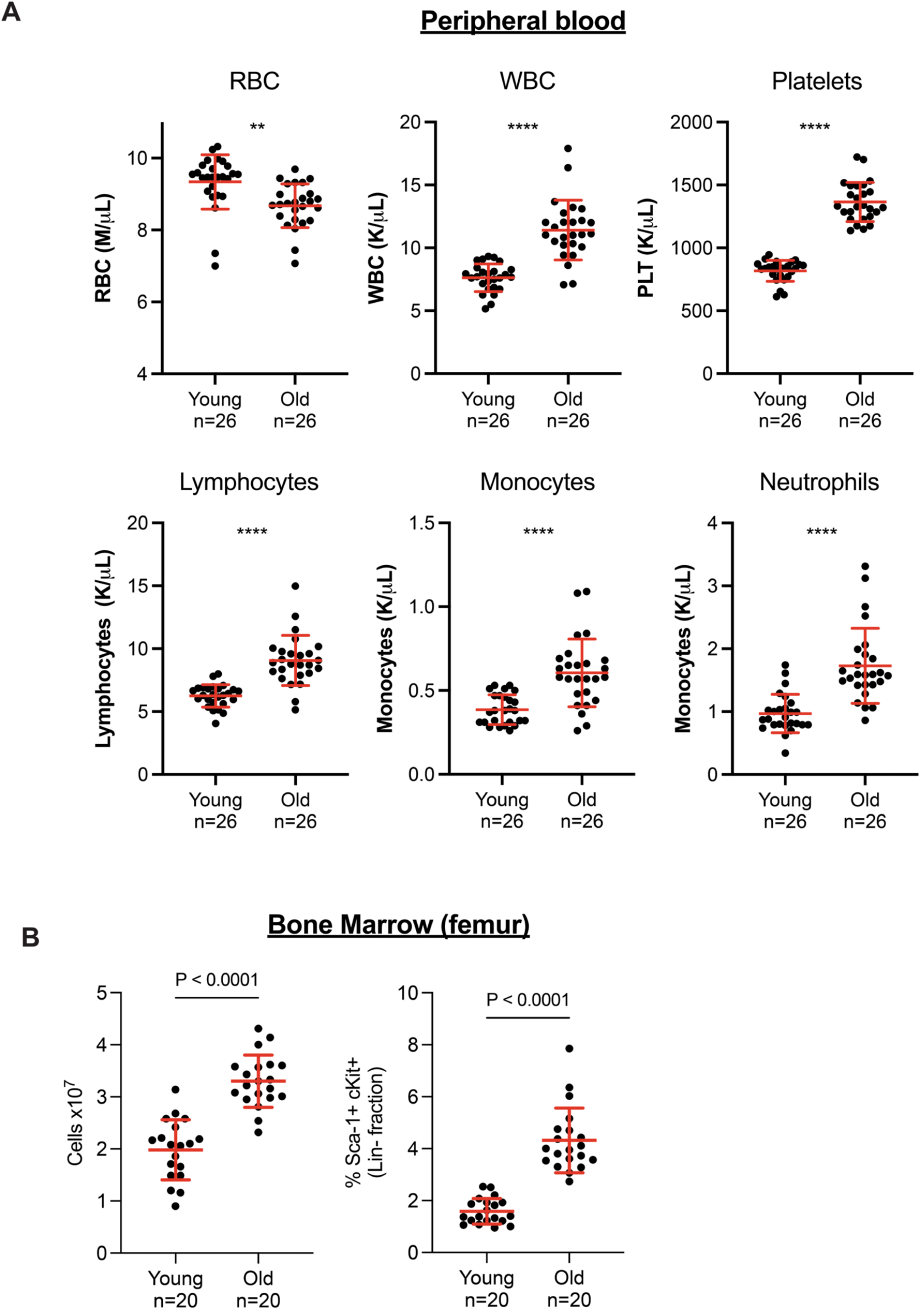
Phenotyping of aged C57BL/6J hematopoietic system. (A) Peripheral blood counts of young (*n* = 26) and old (*n* = 26) C57BL/6J mice. (B) Percent Sca‐1+ cKit+ HSCs via flow cytometry in the lineage depleted (−) fraction of bone marrow isolated from femurs of young and old mice, *n* = 20 for each group. Lineage markers used for depletion are CD5, CD45R (B220), CD11b, Anti‐Gr‐1 (Ly‐6G/C), 7–4, and Ter‐119. For (A, B) *p*‐values were calculated using Welch's *T*‐test. **p < 0.01, ****p < 0.0001, or as denoted.

To determine the cellular landscape of the aged hematopoietic system in mice, we isolated peripheral blood mononuclear cells (PBMCs) and HSCs from 4‐month‐old (young) and 24‐month‐old (old) male C57Bl/6J mice. For peripheral blood, we first performed complete blood counts (*n* = 26 for both young and old groups) showing that aged mice have significant decreases in RBC count, with a concurrent increase in WBCs (lymphocytes, monocytes, and neutrophils) as well as platelet counts (Figure 1A), consistent with previously published historical physiology data from the Jackson Laboratory (www.jax.org). For bone marrow, we extracted total marrow from femurs and total cell counts after RBC depletion, showing there was a significant increase in total bone marrow cellularity in old mice (Figure 1B, *n* = 20 for each age group). We then purified HSPCs using fluorescence activated cell sorting (FACS) after lineage (Lin) marker depletion. These HSPCs were immunophenotyped as a Lin‐cKit+ Sca1+ population, henceforth referred to as LSK+ HSPCs (see Methods and gating strategy found in Figure S1). The percentage of the LSK+ HSPC population was significantly increased from ~1.7% to ~4.2% in old mice (Figure 1B).

### 2.2 Gene Signatures in Aged PBMCs

**FIGURE 2:**
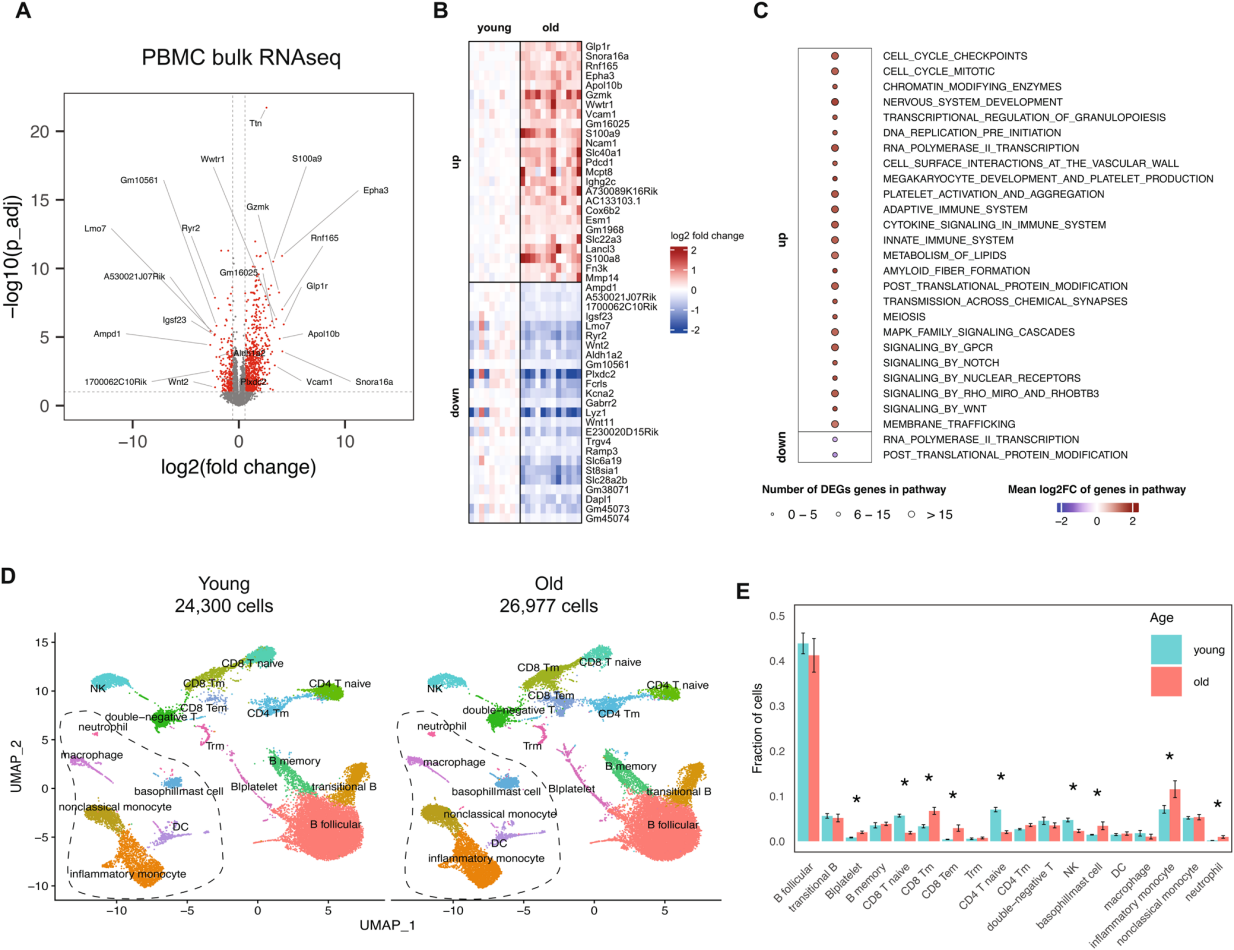
Transcriptomics and scRNAseq of aged peripheral blood mononuclear cells. (A) Volcano plot of differentially expressed genes (DEGs) from bulk RNAseq of PBMCs, young *n* = 10, old *n* = 12. (B) Heatmap of the top 25 significant upregulated and 25 downregulated genes in the bulk RNAseq of A. (C) Significant Reactome pathway enrichment of DEGs, where circle size denotes the number of significant DEGs in the pathway and color denotes the mean log2FC of those genes in the pathway. Only high‐level Reactome pathways with *p*_*adj*_ < 0.01 are shown to limit plot size. (D) UMAP visualization of scRNAseq of combined old (left) and young (right) PBMCs, *n* = 6 mice for each age group. Total cells for each group as denoted. Dotted line surrounds myeloid populations versus lymphoid lineages. (E) Fraction of cells in each cell subtype for young and old samples derived from PBMC scRNAseq data in D. Data represent mean ± s.e.m. *p*‐values calculated using Dirichlet regression, *p < 0.05.

For a deeper characterization of aged mouse PBMCs, we next performed in parallel gene expression profiling using bulk RNAseq (young *n* = 10; old *n* = 12) and cell population heterogeneity profiling using scRNAseq (young *n* = 6; old *n* = 6). We first assessed the bulk RNAseq for global changes in transcription using cutoff of fold change (FC) > 1.5 and *p*_*adj*_ < 0.05. PBMCs from old mice had a significant upregulation of 651 genes and downregulation of 193 genes (Figure 2A, Table S1). The most highly upregulated genes of note in PBMCs are *Glp1r*, *Vcam1*, *Grzmk*, and *Wwtr1* (Taz), and the most down‐regulated genes are *Lyz1*, *Plxdc2*, *Ryr2*, and *Lmo7* (Figure 2B). Unsurprisingly, markers of T cell exhaustion, notably *Pdcd1* (PD‐1), *Tigit*, and *Lag3*, were also among the most upregulated genes (Figure 2B, Table S1). Notably, we also observe Wnt2 and Wnt11 downregulation, possibly leading to decrease in β‐catenin signal for cell proliferation. To validate the RNAseq analysis pipeline, we utilized qPCR using probe‐based arrays. We tested eight up‐regulated and six down‐regulated genes that varied by fold change and expression levels, confirming our RNAseq results for PBMCs are accurate (Figure S2).

To gain insight into the biological functions altered by the aging transcriptome of PBMCs, we performed a hypergeometric test to identify Reactome pathways (Milacic et al. 2024) enriched in genes that are differentially expressed between old and young PBMCs. Overall, 74 pathways were significantly (*p*_*adj*_ < 0.05) enriched in the genes upregulated in the old PBMCs, while only 3 were enriched in the downregulated genes (Table S2). Pathways upregulated with age fell into 5 main themes: regulation of cell proliferation, adaptive and innate immune system, protein homeostasis, various signaling pathways, and cellular senescence (Figure 2C, Table S2). Strikingly, PBMCs show an opposite effect in comparison to most other aged tissues as cell proliferation and protein homeostasis have been shown to be downregulated with aging, while the adaptive and innate immune response is almost universally upregulated across all tissues during aging (Schumacher et al. 2008; White et al. 2015; Benayoun et al. 2019; Shavlakadze et al. 2019, 2023). Cellular senescence, the state of permanent cell cycle arrest, as an upregulated pathway (*p*_*adj*_ = 9.78 × 10^−9^) corroborates previous findings that the circulating immune system undergoes immunosenescence, a proinflammatory state with combined dampened immune function, during aging and not just classical exhaustion (Bektas et al. 2017). For downregulation, the most affected pathway was RNA polymerase II transcription (*p*_*adj*_ = 0.0007) followed by Post‐Translational Protein Modification (*p*_*adj*_ = 0.002).

Since it is well known that circulating cell composition changes with age, we sought to determine the cellular changes in aged PBMCs from C57Bl/6J mice using single cell RNAseq (scRNAseq). We isolated PBMCs from young and old mice (*n* = 6 for each) and performed 10X Chromium platform for each sample. A total of 51,277 cells (24,300 young and 26,977 old) were obtained after quality control (Table S4) and were mapped into 18 clusters based on expression of well‐established markers (Figure S3A, Table S3). UMAP visualization clearly delineated the lymphoid and myeloid sub populations (Figures 2D and S3B). When analyzing the fractions of each population, aged mice had significantly increased fractions of platelets, CD8+ T memory (Tm) and CD8+ T effector memory (Tem), basophil/mast cells, neutrophils, and inflammatory monocytes (Figure 2E). Conversely, and as expected, CD4+ and CD8+ naïve cells significantly decreased with age, likely owing to constant exposure and differentiation into memory or regulatory T cells. These findings in circulating blood cells further validate recent findings from our own group in splenic T cells using ATACseq, showing decreases in naïve T cells with concomitant increases in effector, memory, and exhausted T cells (He et al. 2025). We also observed a significant decrease in natural killer (NK) cells in aged mice, which would contribute to a lack of immediate immune response during aging. Notably, our results are also in agreement with reports from other groups that conducted exhaustive analyses of peripheral blood in both mouse and human, inclusive of centenarians (Tabula Muris Consortium 2020; Karagiannis et al. 2023; Zhu et al. 2023; Filippov et al. 2024).

### 2.3 Gene Signatures in Aged Bone Marrow Hematopoietic Niche

**FIGURE 3:**
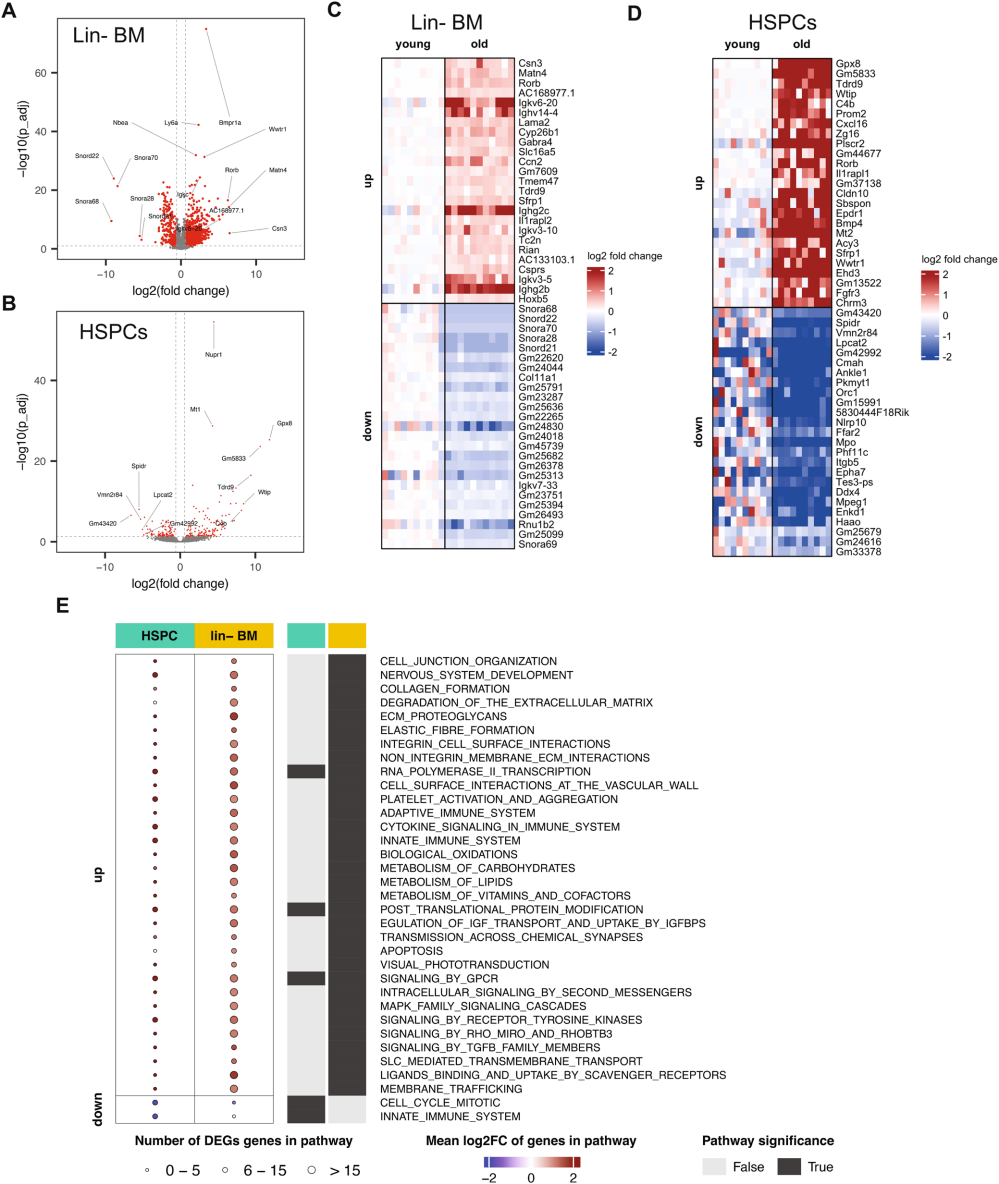
Transcriptomics of bone marrow derived aged hematopoietic stem and progenitor cells. (A) Volcano plot of differentially expressed genes (DEGs) from bulk RNAseq of sorted LSK+ HSPCs, young *n* = 10, old *n* = 10. (B) Volcano plot of differentially expressed genes (DEGs) from bulk RNAseq of Lin‐bone marrow hematopoietic cells, young *n* = 10, old *n* = 11. (C) Heatmap of the top 25 significant upregulated and 25 downregulated genes in the bulk RNAseq of LSK+ HSPCs. (D) Heatmap of the top 25 significant upregulated and 25 downregulated genes in the bulk RNAseq of Lin‐bone marrow cells. (E) Significant Reactome pathway enrichment of significant DEGs for both HSPCs and Lin‐bone marrow cells, where circle size denotes number of significant DEGs in the pathway and color denotes the mean log2FC of those genes in the pathway. Black boxes to the right of each row indicate that the pathway was significant for that compartment. Only high‐level Reactome pathways with *p*_*adj*_ < 0.01 are shown to limit plot size.

Since PBMC characterization only portrays a glimpse of the terminally differentiated blood compartment, we also sought to understand the origins of age‐related dysregulation by analyzing the aged bone marrow niche. We specifically focused on the stem and progenitor cell populations of the hematopoietic system, that is, those populations that lack lineage commitment markers and terminal differentiation markers. To do this, we performed bulk RNAseq on Lin‐bone marrow hematopoietic cells (young *n* = 10; and old *n* = 11) as well as sorted LSK+ HSPCs (young *n* = 10; old *n* = 10), as in Figure S1. Aged Lin‐BM cells showed significant (FC > 1.5 and *p*_*adj*_ < 0.05) upregulation of 1124 genes and down‐regulation of only 361 genes (Figure 3A,C). The large number of significant DEGs is likely due to large shifts in the cell populations within the niche that occur with age. LSK+ HSPCs, however, being a more homogenous population, based on sorting a discreet population, showed more modest but still significant upregulation of 148 genes and down‐regulation of 107 genes (Figure 3B,D; FC > 1.5 and *p*_*adj*_ < 0.05). To validate the RNAseq results, we again utilized qPCR using probe‐based arrays. We tested and confirmed eight up‐regulated and four down‐regulated genes in Lin‐hematopoietic BM cells that varied by fold change and expression levels (Figure S4). In sorted LSK+ HSPCs, we needed to utilize a separate cohort of young and aged mice due to low RNA yields from a low number of cells. Here, we tested six upregulated and four down‐regulated genes (Figure S5); while two genes did not meet significance cut‐off, their trends were confirmed. Additionally, since these were a separate cohort of mice we note that the RNAseq results are robust enough to extrapolate across cohorts. An earlier publication performed proteomic profiling of aged HSPCs (Zaro et al. 2020), where many of our top upregulated genes in aged HSPCs were also increased at the protein level, such as Mt2, Clca3a1, Selp, Slamf1, Enpp5, and Gata2, further supporting the directionality and validity of our dataset. Considering that Lin‐BM includes the HSPC population, we also sought to determine the overlap of differentially expressed genes between the hematopoietic bone marrow cells and stem and progenitor cells. We observe an overlap of 61 up‐regulated genes, notably *Vwf*, *Wwtr1* (Taz), and *Slamf1* (Cd150), *Bmp4*, and *Sfrp1*, and an overlap of only eight down‐regulated genes, notably *Ddx4* (Figure S6). Not surprisingly, both Lin‐hematopoietic BM and HSPCs showed unique top up‐ and down‐regulated genes (Figures 3A–D and S6).

To understand mechanistically what may be dysregulated during aging in stem and progenitor cells, we next input these significantly differentially expressed genes into pathway enrichment to determine which biological functions are most dysregulated. Strikingly, HSPCs were distinctly different from Lin‐BM cells, as these two groups had very few common pathways altered during aging (Figure 3E, Table S2). Common upregulated pathways were that of post‐translation modification, RNA Pol II transcription, cytokine signaling, and platelet aggregation. The up‐regulated genes in Lin‐hematopoietic BM uniquely displayed significant enrichment for common age‐related signatures normally decreased during aging, such as collagen formation and ECM matrix function. Aged HSCs had enrichment in down‐regulation of cell cycle and mitosis pathways, suggesting decreased proliferative capacity in the niche. The most notable pathway in aging, innate immune activation, was up‐regulated in aged Lin‐BM; however, this was strikingly down in the HSPCs. Of all tissues and systems profiled by our group and others, this is one of very few cell/tissue populations that displays age‐regulated downregulation of the innate immune pathway (Shavlakadze et al. 2019; Morsy et al. 2026).

**FIGURE 4:**
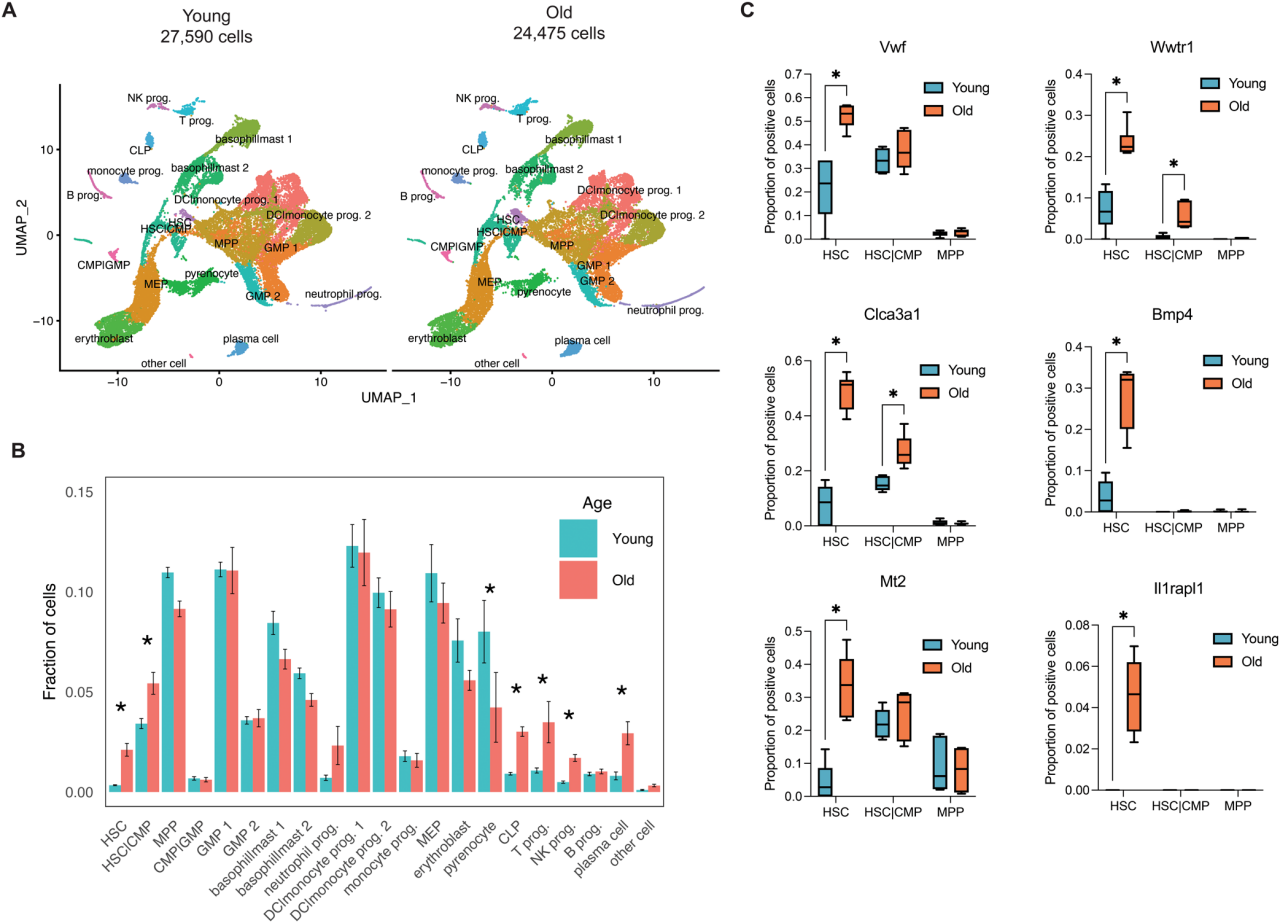
scRNAseq of aged Lin‐bone marrow progenitor cells. (A) UMAP visualization of scRNAseq of young (left) and old (right) isolated Lin‐bone marrow cells, *n* = 6 mice for each age group, total cells for each group as denoted. (B) Fraction of cells in each cell subtype for young and old samples derived from lin‐scRNAseq data in A. Data represent mean ± s.e.m. *p*‐values calculated using Dirichlet regression, *p < 0.05. (C) Proportion of cells expressing the indicated gene in old versus young across HSC, HSC|CMP, and MPP cell types. Box and whiskers denote quartiles of each group (*N* = 6 for both young and old). *p*‐values calculated using unpaired *t*‐test, *p < 0.05.

To further characterize the bone marrow niche, we performed scRNAseq on the Lin‐enriched bone marrow. We isolated bone marrow and performed lineage marker depletion on young and old mice (*n* = 6 for each) and again performed 10X Chromium platform for each sample. A total of 52,065 cells (27,590 young and 24,475 old) were obtained after quality control measures (Table S5) and were mapped into 21 different clusters using expression of well‐established markers (Figure S7A, Table S3). UMAP visualization of the lineage marker depleted BM cells identified 21 clusters in young and old samples (Figures 4A and S7B). While we did not immediately observe any unique cell populations appear or disappear with age, we did, however, observe significant differences in the proportion of classified cell types (Figure 4B). We observe significant increases in CLP (common lineage progenitor), T progenitors, NK progenitors, and plasma B cell populations and a significant decrease in pyrenocytes, that is, cells with extruded nuclei derived from erythroblasts that are encased in a plasma membrane (Figure 4B). Notably, the HSC (defined here as Slamf1/Cd150+, Ly6a/Sca‐1+, cKit+) and HSC/CMP (common myeloid progenitor) pool also significantly increase with age, confirming our prior FACS data (Figures 1B and 4B).

Next, we asked if targets identified in our bulk RNAseq are concordant in the scRNAseq dataset. To do this, we visualized the proportion of *Vwf*, *Wwtr1*, *Clca3a1*, *Bmp4*, *Mt2*, and *Il1rapl1* positive cells in both young and old HSC subsets. Indeed, we observed a significant increase of the proportion of HSC populations that were positive for these markers (Figure 4C). Additionally, these markers displayed increased expression in HSCs as well (Figure S4B). Strikingly, we observed that in the HSC population there exist two populations of HSCs, those that are *Vwf* + *Wwtr1* + *Clca3a1* + and a smaller population lacking all three of these markers, similar to those found in young HSCs (Figure S8A). This is consistent and replicates prior findings that Taz and its downstream target Clca3a1 both mark an “aged” HSC subset (Kim et al. 2022).

**FIGURE 5:**
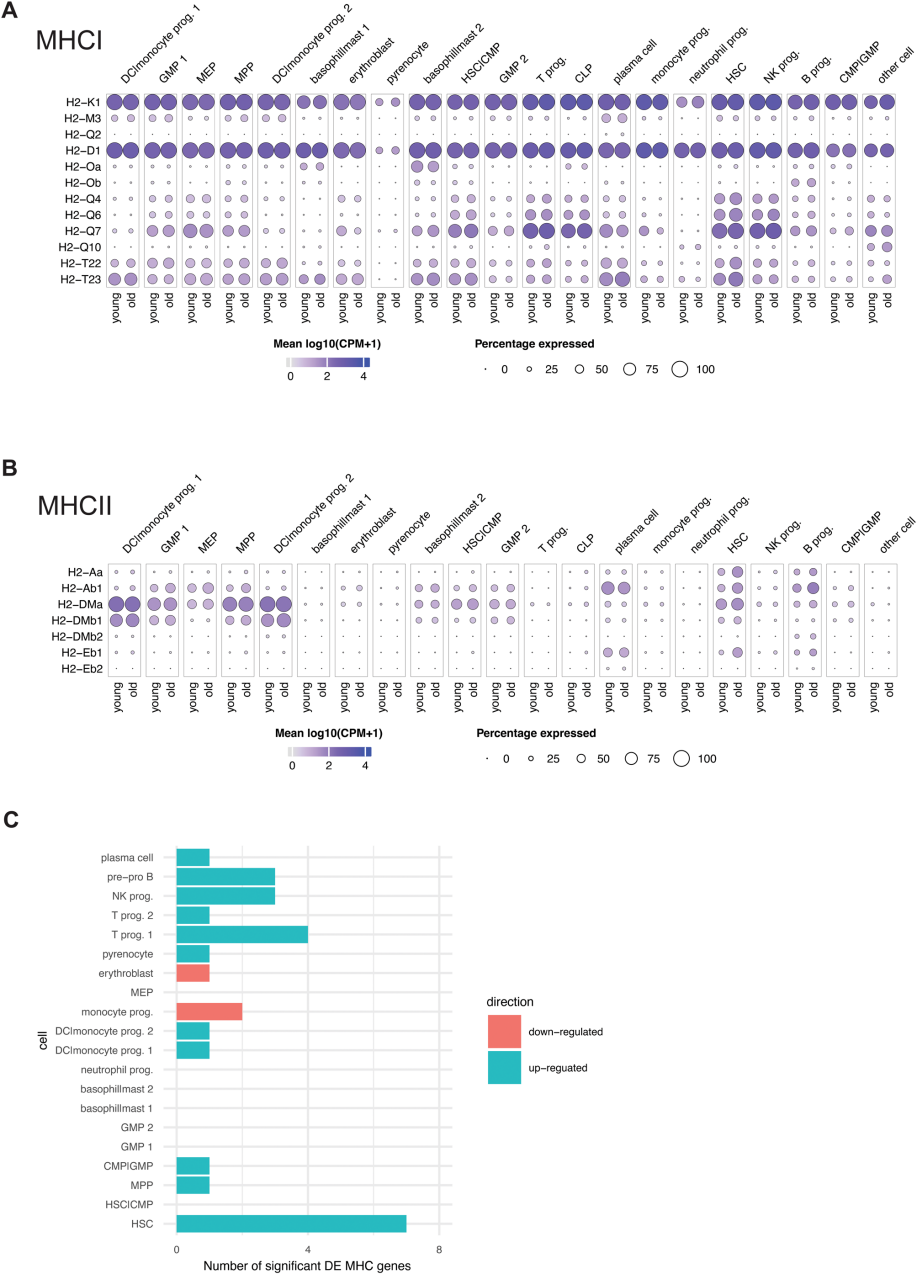
Aging predominantly increases MHC gene expression in hematopoietic stem and progenitor cells. (A) Dot plots representing the proportions of cell subtypes in scRNAseq expressing MHC class I in young and old. Color denotes mean log_10_(CPM + 1). (B) Dot plot representing MHC class II expression as in (A). (C) Bar plot representing the number of significantly differentially expressed MHC loci in young versus old.

Next, we sought to evaluate MHC‐I and MHC‐II expression in our lin‐BM scRNAseq data. Recently published data highlighted that increased production of the MHC‐II complex on myeloid cells can alter their function, concurrent with their increased presence in the aged bone marrow niche (Barman et al. 2022). To assess this in our data, we plotted MHC expression versus the respective MHC loci for each young and old cell population. MHC‐I loci *H2‐K1* and *H2‐D1* are almost ubiquitously expressed across all cell populations, except for pyrenocytes (Figure 5A). For MHC‐II, which is classically restricted to myeloid lineage, we confirm that DC/monocyte populations have the highest percentage expression of *H2‐DMa* and *H2‐DMb1*, followed by HSC, MPP, CMP, and GMP populations (Figure 5B). Differential expression of MHC loci revealed that most populations have at most 1–3 alleles differentially expressed. Interestingly, however, HSCs had seven out of 19 MHC loci significantly differentially expressed (Figure 5C), suggesting that HSCs have the most MHC changes with age. One of these loci, *H2‐T23* (the mouse homolog HLA‐E), increased in aged HSCs and has been implicated as a marker of senescent cells in both mouse and humans (Pereira et al. 2019).

### 2.4 Integration of scRNAseq From PBMC and Bone Marrow Hematopoietic Niche

**FIGURE 6:**
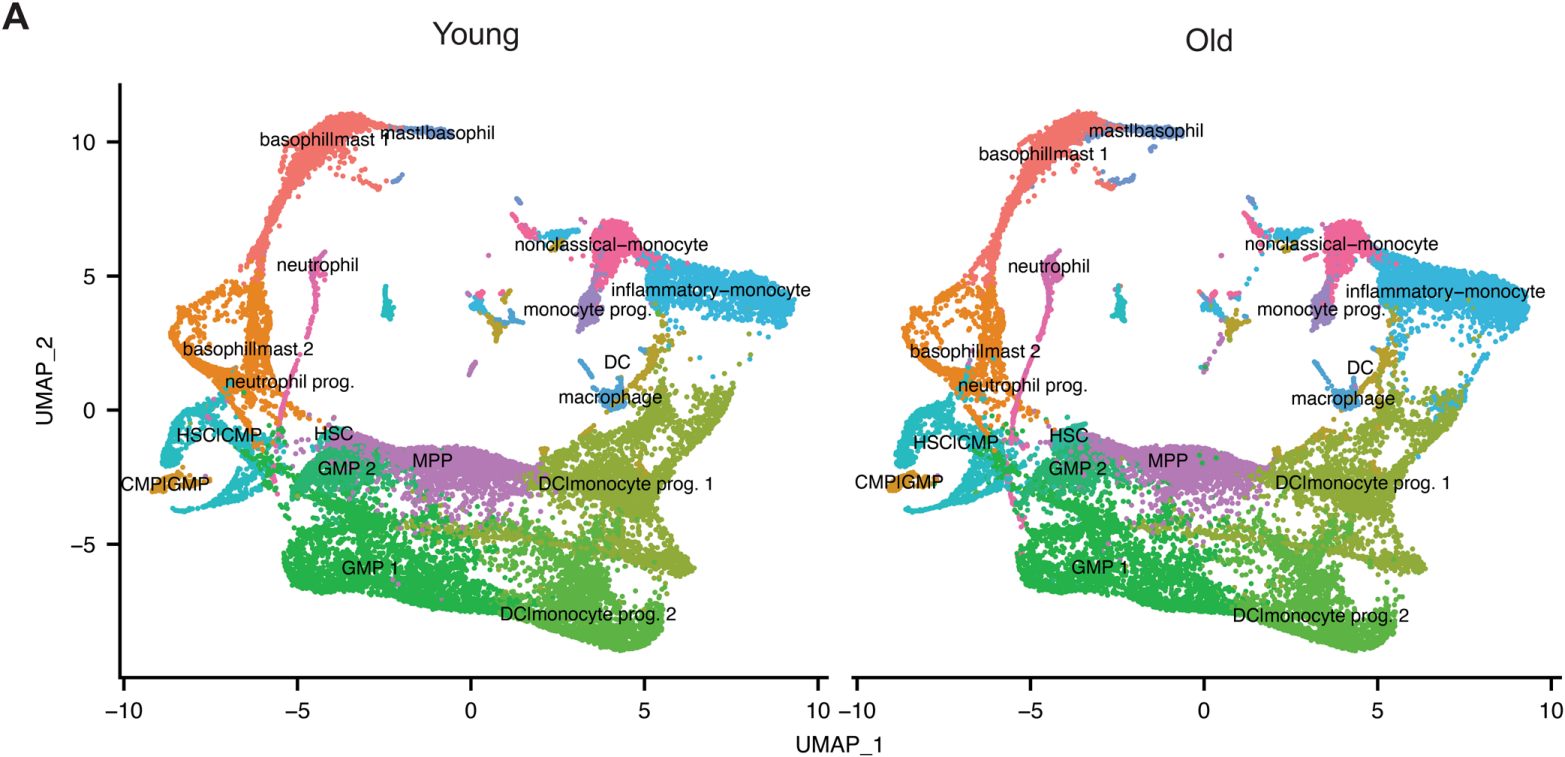
Integration of myeloid‐lineage populations from PBMC and Lin‐bone marrow hematopoietic cells. (A) UMAP visualization of the integrated scRNAseq data of myeloid lineage specific PBMC cells and Lin‐bone marrow hematopoietic cells combined from Young (left) and Old (right). Most pluripotent population trajectory begins at HSC (center left of UMAP).

To explore the interplay between the age‐related changes occurring in the hematopoietic system, we integrated both scRNAseq datasets from the PBMCs and from the lineage marker depleted BM hematopoietic cells. We were able to integrate these datasets and retain cell trajectories as the scRNAseq samples were derived from the same animals. We focused on visualization of progenitor and myeloid lineage cells from the two compartments under the same UMAP (Figure 6). Since we did not perform scRNAseq analysis of thymus or spleen, we did not run integrative analysis on lymphoid lineage cells. As shown in Figure 6, bone marrow derived myeloid cells and PBMC derived myeloid cells of the same lineage were grouped together. This was most obvious in the neutrophil progenitors of the BM and the neutrophils of PBMC, the two basophil|mast clusters 1 and 2 of bone marrow and the mast|basophil cluster of PBMC, and the DC|monocyte progenitor 1 cluster of BM and the DCs, macrophages, and the inflammatory monocytes of PBMC (Figure 6). While we observed the expansion of many of these cell types in the PBMC of aged mice (Figure 2E), there was not a significant expansion of their respective progenitors in the bone marrow of old mice (Figure 4B). However, we observed more overlaps in the old mice between the basophil|mast 1 cluster in bone marrow and the basophil|mast cluster in PBMC, as well as between the DC|monocyte progenitor 1 cluster in bone marrow and the inflammatory monocyte cluster in PBMC (Figure 6). This overlap in the aged hematopoietic system suggests that progenitors themselves can be poised for subsequent inflammatory trajectories, likely contributing to immune dysfunction.

## 3 Discussion

Our study on the transcriptomic and cellular composition of the hematopoietic compartment provides insight into the complex dynamics that occur across diverse blood lineages during aging. By surveying gene expression changes across three different bulk populations (PBMCs, bone marrow progenitors, and HSPCs) we find widespread alterations that are consistent with our prior work in other tissues, whereby inflammatory pathways are upregulated, including genes consistent with the innate immune system (Shavlakadze et al. 2023). We also show that HSPCs have a gene expression signature that is clearly distinct in comparison to multipotent progenitors and terminally differentiated blood cells, suggesting a unique regulation mechanism that distinguishes these cells from their daughter cells. By integrating myeloid‐lineage cells from PBMC and Lin‐BM scRNAseq data, we find that certain myeloid progenitor populations, such as DC|monocyte progenitors—and in particular inflammatory macrophages—overlap and contribute to more deleterious states during aging, for example, inflammatory macrophages.

In addition to bulk RNAseq, we also utilized scRNAseq to identify 39 distinct blood cell types across both PBMC and bone marrow isolations. We find consistent myeloid skewing during aging, accompanied by a switch from naïve to memory/regulatory T cells in lymphoid lineage. Furthermore, we identify specific gene signatures of “aged” HSCs marked by highly upregulated *Vwf*, *Wwtr1*, and *Clca3a1* expression, concordant with previous findings (Kim et al. 2022). By taking a multi‐pronged and well‐powered approach, we hope to have provided a comprehensive resource to guide insight into the aged hematopoietic system.

The constant exposure of an organism and its immune system to various antigens over the course of a lifetime is a likely cause of the observed increase in memory T cells and concurrent reduction in naïve T cell population during aging. Recent evidence suggests that this process can, in part, be modulated by inhibiting the mTORC complex using rapamycin or its analogs, which have been shown to be the one bona fide aging intervention that mechanistically performs similarly across eukaryotes (Mannick et al. 2014; Mannick and Lamming 2023). This effect was largely attributed to the rapalog effect on reducing PD‐1+ cells in circulation. Our data further demonstrate that both PD‐1 and Grzmk are upregulated almost 10–20‐fold in PBMCs, in line with evidence from aged splenic T cells from our group, and others (Mogilenko et al. 2021; He et al. 2025), and thus rapalogs may suppress pro‐inflammatory states in aged T cells. Given the evidence that PD‐1+ Grzmk+ T cells are increased in circulation, it may serve a useful target in depletion strategies to reduce pro‐inflammatory T cells in aging.

Wnt signaling itself has been associated with aging through findings in senescent cells via the finding that loss of Wnt2 is capable of inducing a senescent phenotype such as SA‐β‐gal and heterochromatic foci (Ye et al. 2007; Hofmann et al. 2014). Intriguingly, in our dataset we find a decrease in both Wnt2 and Wnt11 with age in PBMCs (see Figure 2B). Additionally, we find that TAZ (*Wwtr1*), part of the YAP/TAZ (Hippo) pathway that regulates the β‐catenin destruction complex (Azzolin et al. 2014), is highly upregulated across PBMCs, Lin‐BM, and in HSPCs. Since Wnt signaling and the downstream Hippo pathway can regulate proliferation, it would be of interest to dissect whether Wnt agonism can help to overcome immunosenescent blood phenotypes, and to explore the interplay between the PBMC and bone marrow niches with respect to Hippo signaling.

In addition to these findings, we find that in aged lin‐bone marrow hematopoietic cells a large enrichment of upregulated genes is involved in cytokine signaling and innate and adaptive immune response, much in line with functional studies showing aged bone marrow acquires a pro‐inflammatory niche, likely through IL‐1 (Mirantes et al. 2014; Mitchell et al. 2023). Interestingly, however, few downregulated genes enriched for a specific pathway, but we did observe that many of these genes were from a class of small nucleolar RNAs (snoRNAs). SnoRNAs function mainly to modify ribosomal RNAs by addition of pseudouridylation or 2′‐O‐methylation (Huang et al. 2022), however, to date little is known about the alterations of these modifications during aging. Hence, the aged bone marrow niche may serve as a good system to test perturbations in snoRNAs and their effect on ribosomal biogenesis.

Using the scRNAseq data we were able to map MHC class I and II expression across stem and progenitor cells in the bone marrow niche, showing that HSPCs had the most differentially expressed loci. Many of these are MHCII and as such may serve as one indicator of skewing to myeloid lineage. Previous studies have shown that bone marrow derived monocytes have higher expression of MHCII (Barman et al. 2022), however, higher expression of classical MHCII does not rescue the lack of macrophage function as evidenced by defective phagocytosis, efferocytosis, and antigen display function (Frisch et al. 2019). It would be important to test if monocytes derived from these MHCII+ high expressing HSCs are the root cause for the impaired phagocytosis and efferocytosis process observed in aged bone marrow derived macrophages.

Based on our findings in our scRNAseq data from a separate “aged” HSC population marked by high *Vwf + Wwtr1* + *Clca3a1+* expression, and earlier findings by Kim et al. (Kim et al. 2022), it is plausible to posit that clearance of these cells may rejuvenate the aged immune system. Indeed, recent evidence showed that antibody mediated clearance of myeloid skewed HSCs using the markers CD62p, NEO1, or CD150 alone or in combination with cKIT and/or CD47 was able to restore HSCs with balanced lymphoid and myeloid potential in aged mice (Ross et al. 2024). Moreover, this study showed that after myeloid skewed HSC depletion, the immune system was able to restore functional immunity in aged mice to a vaccination and infection (Ross et al. 2024), much in line with evidence from human clinical trials of mTORC1 inhibition (Mannick et al. 2014, 2018). Our results in old PBMCs also show that there is a marked increase in exhausted T cells and possible decline in B cell functionality, thus coupling aged HSC depletion in conjunction with exhausted T (Grzmk+, PD‐1+) or B cell depletion to allow for more naïve or immature T and B cells could drastically improve an aged immune system. Further research into human therapeutics and the potential for these conditioning regimens needs to be assessed, but the implications on human health after immune rejuvenation could be far‐reaching.

## 4 Materials and Methods

### 4.1 Animals and Procedures

All procedures involving animals were approved by the Institutional Animal Care and Use Committee of Regeneron Pharmaceuticals. Male C57Bl/6J mice were purchased from the Jackson Laboratory and maintained in animal holding facilities for a minimum of 4 weeks prior to tissue collection under specific pathogen free (SPF) conditions. Mice were housed under 12‐h light/dark cycles and provided food (Picolab Rodent Diet 20) and water ad libitum. For complete blood count analysis, mice were bled via submandibular vein, ~50 μL, one week prior to time of tissue harvest. Blood was collected in K3 EDTA tubes to prevent clotting prior to analysis on the Oxford Genesis CBC system.

### 4.2 Isolation of PBMCs

Mice were euthanized and up to 1 mL of terminal blood collected into K3 EDTA tubes. Mononuclear cells were isolated using Lymphoprep and SepMate‐15 tubes (StemCell Technologies) according to the manufacturer's protocol. Purified PBMCs were washed twice using 1× D‐PBS + 2% FBS, and then subjected to either DNA isolation, RNA isolation, or single cell RNA sequencing.

### 4.3 Isolation of Lin^−^ and Hematopoietic Stem Cells

Femurs from euthanized mice were harvested and processed according to previously published protocols (Amend et al. 2016). Briefly, bone marrow was spun out of whole femurs and resuspended in 1× D‐PBS containing 0.5% FBS and 2 mM EDTA (PBE) buffer. Whole marrow was then subjected to RBC lysis using ACK lysis buffer (Gibco) and then strained through 70 μm filters and pelleted by centrifugation. RBC‐depleted marrow cells were then used for lineage negative enrichment using Lineage Cell Depletion Kit (130–090‐858, Miltenyi Biotec) according to the manufacturer's protocol, using LS columns on QuadroMACS separator (Miltenyi Biotec). Lineage markers used for depletion are: CD5, CD45R (B220), CD11b, Anti‐Gr‐1 (Ly‐6G/C), 7–4, and Ter‐119, such that any cell positive for these markers will be selected against and excluded from the lineage negative fraction (Solomon et al. 2020). Samples from all animals were kept separate and not combined for any steps. Lineage negative cells were then washed and centrifuged. For single cell RNAseq, cells were resuspended at 1000 cells/μL. Lin‐cells for bulk RNAseq were resuspended in TRIzol for RNA isolation. Lin^−^ cells for HSC isolation were resuspended in a staining cocktail of anti‐Ly6A/E‐FITC (Sca1, BioLegend), anti‐CD117‐PE (cKit, BioLegend), streptavidin‐APC (BioLegend), mouse FcR blocking reagent (Miltenyi Biotec) in PBE buffer for 15 min then washed and pelleted. Cells were resuspended in 200uL PBE + DAPI and then sorted based on staining using FACSAria Fusion cell sorter (BD Biosciences). The gating strategy can be found in Figure S1.

### 4.4 RNA Sequencing

Ribosomal RNA‐depleted RNA‐seq libraries were generated using QIAseq FastSelect—rRNA HMR (QIAGEN) with the KAPA RNA HyperPrep Kit (Roche Sequencing). Starting material was 100 ng of RNA dependent on availability per tissue type. RNA fragmentation was done at 85°C for 6 min. cDNA was ligated with 300 nM xGen Dual Index UMI Adapters (Integrated DNA Technologies). Ligated cDNA libraries were amplified using 14 PCR cycles, also respective to RNA input. Sequencing of the resulting libraries was done on NovaSeq 6000 (Illumina) using a 2 × 76, paired‐end sequencing recipe. Raw reads were aligned to mouse genes according to GRCm38 and Ensembl 100. Of note, one old lin‐BM sample failed library prep and was excluded from subsequent sequencing and analysis.

### 4.5 Low Input RNA Sequencing

cDNA synthesis was performed directly on lysed HSCs using the SMART‐Seq v4 Ultra Low Input RNA Kit (Takara Bio). From cDNA, libraries for sequencing were prepared using the Nextera XT DNA Library Prep method (Illumina) and sequencing of the final libraries was performed on the NovaSeq 6000 (Illumina) by multiplexed paired‐read run with 2 × 76 cycles. Of note, two old HSC preps failed library prep and thus were excluded from any sequencing and analysis.

### 4.6 Single Cell RNA‐Sequencing

Single cells suspended in PBS with 0.04% BSA were loaded, 10 K cells per lane, on a Chromium Controller (10× Genomics). RNA‐seq libraries were prepared using the Chromium Next GEM Single Cell 3′ Kit, v3.1 (10× Genomics) with single‐index barcoding, according to the manufacturer's guidelines. Paired‐end sequencing was performed on the Illumina NovaSeq 6000 for RNA‐seq libraries (Read 1 28‐bp for UMI and cell barcode, Read 2 80‐bp for transcript read, with 8‐bp i7 and 0‐bp i5 reads). Cell Ranger Single Cell Software Suite (10X Genomics, 6.1.1) was used to perform sample de‐multiplexing, alignment, filtering, and UMI counting. The mouse GRCm38 genome assembly and Ensembl100 gene model for mouse were used for the alignment.

### 4.7 Bulk RNA‐Seq Analysis

We run DESeq2 (Love et al. 2014) on the raw counts of gene expression levels to identify differentially expressed genes between old and young samples. After running DESeq2, we filtered out genes that have low counts. For each gene, we identified samples where the gene has less than 10 reads. The gene was filtered out if its read count was low in more than 20% of the old (young) samples, if the gene had higher expression in the old (young) in the result of DESeq2. The *p* values of the remaining genes were adjusted for multiple comparisons by the Benjamin–Hochberg procedure. Genes with an adjusted *p*‐value < 0.05 and fold change > 1.5 were considered significant.

To identify biological processes associated with the differentially expressed gene, we downloaded the Reactome pathway annotations (Gillespie et al. 2022) from MSigDB (Subramanian et al. 2005). We performed hypergeometric test to identify pathways that were enriched in the differentially expressed genes, and the up‐regulated genes and the down‐regulated genes were analyzed separately. Pathways that overlapped with less than 10 differentially expressed genes were ignored. The rest were adjusted for multiple comparison by the Benjamin–Hochberg procedure, and an adjusted *p*‐value < 0.05 was considered significant.

### 4.8 Single Cell RNA Sequencing Analysis

Raw scRNA data were processed by Cellranger by using the default parameters to filter out non‐cell barcode. The filtered data were processed by Seurat4 for clustering (Hao et al. 2021).

For lin‐bone marrow samples, we filtered out cells with less than 500 genes or with 15% or more UMIs from mitochondrial genes. Table S5 summarizes the numbers of the cells before and after the filtering. We used the reciprocal PCA methods to integrate samples. We picked 2000 highly variable genes and 50 PCs for integration. One young sample and one old sample with relatively good quality (samples s4 and s12, Table S5) were chosen as the reference of integration. After integration, cells were clustered with 30 PCs by using the default Louvain algorithm with a resolution of 0.2. The cell type of each cluster was identified based on markers in Table S3. The marker genes of each cell type were identified by the function “FindAllMarkers” of Seurat4, with the default parameter values. We used fold change > 2 and adjusted *p*‐value < 0.05 to call marker genes. The differentially expressed genes in each cell type between old and young mice were identified by the function “FindMarkers” with the default parameter values, and by fold change > 1.5 and adjusted *p*‐value < 0.1.

For PBMC, we filtered out cells with less than 1000 genes or with 15% or more mitochondrial UMIs. Table S4 summarizes the numbers of the cells before and after the filtering. To integrate the PBMC samples, we picked 5000 highly variable genes and 50 PCs for integration. One young sample and one old sample with relatively good quality (samples s3 and s11, Table S4) were chosen as the reference for integration. Cells were clustered with 25 PCs under a resolution of 0.3. Markers used to identify cell types were listed in Table S3. To identify additional subtypes of T cells, the Cd4/8 T cells, Trm, and double‐negative T cells from the initial clustering were pooled and clustered again using 30 PCs under a resolution of 0.7. The marker genes of each cell type and the differentially expressed genes in each cell type were identified by the same procedures as above.

To identify significant changes in the composition of lin‐bone marrow and PBMC between young and old mice, we performed Dirichlet regression (Maier 2014), which accounts for the constraint that the fractions of all cells must sum to one. We used the R package DirichletReg and set the base to DC|monocyte prog. One in lin‐bone marrow and to B follicular in PBMC because both cells show high and apparently stable abundances between young and old mice.

We integrated myeloid‐lineage cells of the bone marrow and PBMC samples. We pooled bone marrow cells of DC|monocyte progenitors 1/2, basophil|mast 1/2, monocyte progenitor, neutrophil progenitor, GMP1/2, CMP|GMP, HSC|CMP, MPP, HSC, and PBMC cells of inflammatory monocyte, nonclassical monocytes, mast|basophil, DC, macrophage, neutrophil. We split the pooled cells by the animals into 12 Seurat objects, and integrated the 12 Seurat objects with the CCA method. We used 2000 highly variable genes for integration. The integrated data was projected onto a 3‐dimensional UMAP by using 30 PCs and used the first two UMAP dimensions for visualization. We retained the original identities of cells and did not recluster the cells.

### 4.9 qPCR

Total RNA samples were reverse transcribed into cDNA using SuperScript IV VILO kit (ThermoFisher). First strand cDNA was then used for amplification using Taqman Fast Advanced Master Mix (ThermoFisher) with the probes listed in Table S6. Calculations were performed using the ΔΔCT method normalizing to either Polr2a or 18s rRNA as indicated, with biological replicates represented as the mean of technical triplicates. Significance was determined using Welch's *T* test as indicated.

### 4.10 Statistics

Statistical analyses for RNAseq were performed as indicated above for analysis. All other datasets were analyzed using GraphPad Prism software v10. *p*‐values were calculated as indicated and significance value cutoff set at *p* < 0.05 for all tests.

## Author Contributions

R.R.W., T.S., and D.J.G. conceptualized the study. R.R.W., M.W., and A.S. performed animal dissections and isolation of various blood cell compartments, including flow cytometry. C.A., N.N., and M.N. performed library preparation for bulk RNAseq and single cell RNAseq. R.R.W., K.X., and Y.B. performed bioinformatic analysis and analyzed data. R.R.W. and D.J.G. supervised the research. R.R.W. wrote the manuscript with input from all other authors.

## Funding

The authors have nothing to report.

## Conflicts of Interest

This study was sponsored by Regeneron Pharmaceuticals Inc. All authors are employees of Regeneron and may hold stock and/or stock options in the company.

## Supporting information


**Figure S1:** Flow cytometry gating strategy to quantify and isolate Lineage‐Sca1+ cKit+ HSPCs.

**Figure S2:** qPCR validation of selected upregulated (A) and downregulated (B) genes in PBMCs. Values were calculated using DDC_T_ method and normalization done to Polr2a. Biological replicates represent the mean of technical triplicate values. *p*‐values were calculated using Welch's *T*‐test. ** *p* < 0.01, *** *p* < 0.001, ****p < 0.0001.

**Figure S3:** scRNAseq markers from PBMCs. (A) Markers and their expression values for each cell population identified from PBMCs. (B) Individual UMAP plots for each animal (Young *n* = 6, Old *n* = 6).

**Figure S4:** qPCR validation of selected upregulated (A) and downregulated (B) genes in bulk Lineage‐bone marrow stem and progenitor cells (HSPCs). Values were calculated using DDC_T_ method and normalization done to Polr2a. Biological replicates represent the mean of technical triplicate values. *p*‐values were calculated using Welch's *T*‐test. ** *p* < 0.01, *** *p* < 0.001, ****p < 0.0001.

**Figure S5:** qPCR validation of selected upregulated (A) and downregulated (B) genes in sorted LSK+ HSCs. A separate cohort was used for validation due to limited numbers of HSCs obtained from a single animal. Values were calculated using DDC_T_ method and normalization done to 18s rRNA. Biological replicates represent the mean of technical triplicate values. *p*‐values were calculated using Welch's *T*‐test. * *p* < 0.05, ** *p* < 0.01, *** *p* < 0.001.

**Figure S6:** Venn diagrams of the overlap of significant upregulated (A) and downregulated (B) DEGs from Lin‐BM and HSPCs.

**Figure S7:** scRNAseq markers from Lin‐bone marrow stem and progenitor cells. (A) Markers and their expression values for each cell population identified from Lin‐BM cells. (B) Individual UMAP plots for each animal (Young *n* = 6, Old *n* = 6).

**Figure S8:** Characterization of markers of aged HSCs. (A) Zoom‐in on HSC, showing the expression of Vwf, Wwtf1, and Clca3a1. Color denotes the natural logarithm of CPM + 1 of gene expression calculated using NormalizeData of Seurat. (B) Violin plots of the gene expression Log10(CPM + 1) of the selected genes across HSC, HSC|CMP, and MPP cell types as show in Figure 4C.

**Table S1:** List of significant differentially expressed genes in young versus old mice from HSPC, Lin‐bone marrow, and PBMCs.

**Table S2:** List of significant REACTOME pathways from differentially expressed genes.

**Table S3:** Markers used to define populations from scRNAseq.

**Table S4:** scRNAseq quality control metrics for PBMCs.

**Table S5:** scRNAseq quality control metrics for Lin‐bone marrow hematopoietic cells.

**Table S6:** List of primers and probes used for qPCR validation.

## Data Availability

The data that support the findings of this study are openly available in GEO at reference number GSE310923.
